# Catheter based left atrial appendage closure in-hospital outcomes in Germany from 2016 to 2020

**DOI:** 10.1007/s00392-023-02299-w

**Published:** 2023-09-12

**Authors:** Alexander Maier, Klaus Kaier, Timo Heidt, Dirk Westermann, Constantin von zur Mühlen, Sebastian Grundmann

**Affiliations:** 1 Department of Cardiology and Angiology, Faculty of Medicine, University Heart Center Freiburg-Bad Krozingen, University of Freiburg, Hugstetter Straße 55, 79106 Freiburg, Germany; 2 Institute of Medical Biometry and Statistics, Faculty of Medicine, University of Freiburg, Freiburg, Germany

**Keywords:** LAA closure, Left atrial appendage, In-hospital outcome, Germany

## Abstract

**Background:**

New and refined catheter based left atrial appendage (LAA) closure devices have been introduced in the past decade. The procedure can be performed using either an endocardial occlusion device or an epicardial loop stitch. We aimed to analyzed recent procedural safety.

**Methods:**

Catheter based LAA closures were identified in a complete nationwide German dataset via ICD and OPS codes from 2016 to 2020.

**Results:**

From 2016 to 2020, 28,039 endocardial and 213 epicardial occlusions were performed. Numbers of endocardial procedures increased from 5259 in 2016 to 5917 in 2020 (p = 0.020) in 387 centers with shifting of patients’ characteristics towards older age (β = 0.29, p < 0.001), more heart failure (β = 1.01, p < 0.001) and renal disease (β = 0.67, p = 0.001) and without a significant trend for in-hospital safety except more bleeding (β = 0.12, p = 0.05). In-hospital major adverse cardiac and cerebrovascular events (MACCE) or pericardial puncture were independent on center procedure numbers.

The loop stitch procedure was performed in 15 centers. Patients were younger (76.17 ± 8.16 vs. 73.16 ± 8.99, p < 0.001) and had a lower comorbidity index (2.29 ± 1.93 vs. 1.92 ± 1.64, p = 0.005). Adjusted risk difference for pericardial effusion (8.04%; 95% CI 3.01–13.08%; p = 0.002) and pericardial puncture (6.60%; 95% CI 3.85–9.35%; p < 0.001) was higher for the loop stitch procedure, while risk of bleeding (− 1.85%; 95% CI − 3.01 to − 0.69%; p = 0.002), intracerebral bleeding (− 0.37%; 95% CI − 0.59 to − 0.15%; p = 0.001) and shock (− 1.41%; 95% CI − 2.44 to − 0.39%; p = 0.007) was lower. No significant difference was observed for in-hospital MACCE.

**Conclusions:**

Endocardial occlusion was the major catheter based LAA closure procedure in Germany without improvements in in-hospital safety from 2016 to 2020. In-hospital MACCE was independent on endocardial LAAC center volumes. Conclusions on the comparison between the two procedure types must be made cautious as the LAA loop stitch occlusion was utilized limited in a minor number of centers.

**Graphical abstract:**

Catheter based left atrial appendage closure in-hospital outcomes in Germany from 2016 to 2020

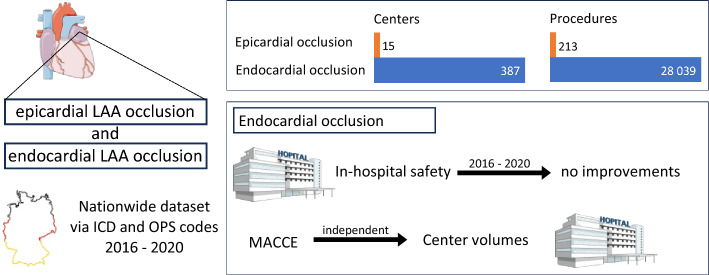

**Supplementary Information:**

The online version contains supplementary material available at 10.1007/s00392-023-02299-w.

## Introduction

The 2020 European Society of Cardiology Guidelines for the diagnosis and management of atrial fibrillation state left atrial appendage closure to be an option for stroke prevention therapy in patients with atrial fibrillation [1–3]. While there is excellent evidence that oral anticoagulation is recommended for stroke prevention in AF patients with CHA_2_DS_2_-VASc score ≥ 1 in men or ≥ 2 in women [1, 4], LAAC may be considered for stroke prevention in patients with known contraindications for such long-term anticoagulant treatment e.g. intracranial bleeding without a reversible cause [5–9].

Beside the surgical option to close the LAA, mainly two catheter based approaches are available today. One approach is LAAC through a permanently implanted endocardial occlusion device (e.g. Watchman, Boston Scientific or Amplatzer Amulet, Abbott). Here, the left atrial appendage is accessed via puncture of the interatrial septum through a venous catheter to place the device in the LAA, resulting in an exclusion of blood flow. The first Watchman and Amplatzer occluders were implanted in 2002. The Watchman occluder received its CE mark in Europe in 2005 [10] and the Amplatzer plug has a CE mark since 2008. After that newer device generations were introduced. FDA approval of endocardial LAA occluders started later beginning with the Watchman device in 2015. In general, higher safety event rates have been reported in real-world analyses [11–15] compared with industry-sponsored studies for these devices [16, 17].

Another, non-FDA-approved option is an epicardial loop stitch around the base of the LAA (Lariat, SentreHeart) [18]. It requires accessing the heart through both transseptal puncture and pericardial access by transthoracic puncture. Lariat was implanted for the first time in 2011 [19] and received the CE mark in 2015 [20]. After the FDA issued a safety alert in 2015 the procedure has been made safer [21, 22] and Lariat XT was released.

Overall, in-hospital complications remain an “Achilles heel” of interventional LAA occlusion. An update of in-hospital safety trends for catheter based LAAC is of particular interest since improvements in device design and new device generations were introduced and entered the clinical routine in the past years. New OPS codes in Germany made it possible to compare in-hospital safety of the permanently endocardial implanted LAA occlusion devices with the epicardial loop stitch procedure around the base of the LAA. In this study, we analyzed procedure numbers of the two available catheter-based LAAC approaches and in-hospital safety trends in the German nation-wide in-patient registry from 2016 to 2020. We additionally compared the in-hospital safety profile of the intracardiac implanted LAA occlusion device procedure with the loop stitch procedure around the LAA base.

## Methods

### Data source

Since 2005, the German Federal Bureau of Statistics (Statistisches Bundesamt, Destatis), through its Research Data Center, provides data on all inpatient stays in Germany. These data are based on inpatient hospital settlements according to the German Diagnosis Related Groups (DRG) system, which is based on fixed charge groups formed on the basis of diagnoses (coded according to ICD-10) and procedures performed [coded according to the German Operation and Procedure Classification (OPS)]. Upon prior request, the Research Data Centre can provide an analysis of your data in the form of fully anonymous, aggregated results, which will be published by the Research Data Centre. If necessary, partial results will be censored. Therefore, investigators only have access to summary results provided by the Research Data Center, an and do not have direct access to individual patient data. Therefore, approval by an ethics committee and informed consent were determined not to be required for our study, in accordance with German law. All of the summary results have been anonymized by the Research Data Centre. In practice, this means that, in order to guarantee data protection, the Research Data Centre censored any information that would allow conclusions to be drawn about an individual patient or a specific hospital.

### Diagnoses and outcomes definitions

We requested data and numbers of patients that underwent the catheter-based transseptal and endocardial LAA occlusion procedure (OPS-code 8-837.s0) and the epicardial loop stitch procedure around the LAA base (8-837.s1) for each year from 2016 to 2020 from the German Research Data Center.

The following patient baseline characteristics were requested: Age, female sex, Charlson comorbidity index (see reference for complete ICD-10 list [23]), arterial hypertension (I10), atrial fibrillation (I480 1 2 9), heart failure NYHA III or IV (I5013 and I5014), coronary artery disease (I25), previous myocardial infarction (I252), previous cardiac surgery (Z951 2 3 4), peripheral vascular disease (I702 8 9 I739), carotid disease (I652), chronic obstructive lung disease (J44), pulmonary hypertension (I27), chronic renal disease (N18), diabetes (E10 1 2 3 4), cancer (ICD C*), surgery (OPS 5*).

The following in-hospital outcome parameters were requested for each year from 2016 to 2020: myocardial infarction (I21), stroke (I63), MACCE (composed of in-hospital mortality, myocardial infarction and stroke), bleeding (transfusion > 5 erythrocyte concentrates, 88800c1—8800cr and 880070—88007e), intracerebral bleeding (I61), pericardial effusion (I312 or I313), pericardial puncture (OPS 81520*), pericardiotomy (OPS 5370*), deep vein thrombosis (I80), tachycardia (I47 R000), shock (R57) and resuscitation (OPS 877*). In-hospital mortality and length of hospital stay were part of DESTATIS’ main set of variables.

### Statistical methods

Categorical variables were presented as n (%). Continuous variables were summarized as mean ± SD. Pearson’s chi-square and t-tests were used to make descriptive comparisons between groups as appropriate. Temporal trends were observed using simple linear regression models.

Since patients were not randomized towards permanently implanted occlusion or a loop stitch procedure, potential confounding factors were taken into account using the propensity score methods. Thereby, inverse probability weighting was applied. The propensity score is defined as the conditional probability of an individual for being in the treatment group, given a group of observed covariates. For the propensity score estimations, we fit logistic regression models with the “teffects ipw” estimation procedures in Stata 17 and controlling for 18 predetermined covariates (all variables listed in Table 1 and Supplement Table 1). To determine the impact of procedure volumes on the endpoints MACCE and pericardial puncture, multivariable logistic regression analyses were performed. For risk adjustment, age, the Charlson Comorbidity Index and procedure volume were included as continuous covariates while all categorical characteristics listed in Table 2 were included as categorical covariates. Cluster-robust standard errors were used to account for the correlation of error terms of patients treated in the same hospital. Two-sided p-values are given, and statistical significance was considered as p-value < 0.05. No adjustments for multiple testing were done. All analyses were carried out using Stata 17 (StataCorp, College Station, Texas, USA).

**Table 1:** Centers per year performing each LAA occlusion procedure from 2016 to 2020

Centers/year	2016	2017	2018	2019	2020
N Centers endocardial implanted device	311	329	353	387	384
N Centers loop stitch around LAA base	15	13	14	11	13

**Table 2:** Baseline characteristics for patients implanted an endocardial LAA occlusion device or loop stitch around the LAA base from 2016 to 2020

Characteristic	Total procedure, n = 28,252	endocardial implanted LAA occlusion device, n = 28,039	loop stitch around the LAA base, n = 213	p
Age (mean ± SD)	76.15 ± 8.16	76.17 ± 8.16	73.16 ± 8.99	< 0.001
Women	11,047 (39.10%)	10,952 (39.06%)	95 (44.60%)	ns
Charlson Comorbidity Index	2.28 ± 1.78	2.29 ± 1.93	1.92 ± 1.64	0.005
Arterial hypertension	16,756 (59.31%)	16,613 (59.26%)	143 (67.14%)	ns
Atrial fibrillation	27,420 (97.06%)	27,216 (97.06%)	204 (95.77%)	ns
CHA_2_DS_2_-VASc (mean ± SD)	4.00 ± 1.41	4.01 ± 1.41	3.79 ± 1.46	0.022
Heart failure NYHA III or IV	5119 (18.12%)	5089 (18.15%)	30 (14.08%)	ns
Coronary artery disease	12845 (45.47%)	12,752 (45.48%)	93 (43.66%)	ns
Previous myocardial infarction	2200 (7.79%)	2193 (7.82%)	7 (3.29%)	0.014
Previous cardiac surgery	3474 (12.30%)	3460 (12.34%)	14 (6.57%)	0.011
Peripheral vascular disease	1998 (7.07%)	1988 (7.09%)	10 (4.69%)	ns
Carotid disease	483 (1.71%)	481 (1.72%)	1–3 (≤ 1.41%)	–
COPD	3519 (12.46%)	3022 (10.78%)	16 (7.51%)	ns
Pulmonary hypertension	2351 (8.32%)	2339 (8.34%)	12 (5.63%)	ns
Chronic renal disease	10,995 (38.92%)	10,921 (38.95%)	74 (34.74%)	ns
Diabetes	8897 (31.49%)	8838 (31.52%)	59 (27.70%)	ns
Cancer	587 (2.08%)	585 (2.09%)	1–3 (≤ 1.41%)	–

*LAA* Left atrial appendage, *SD* standard deviation, *NYHA* New York Heart Association, *COPD* Chronic obstructive pulmonary disease

## Results

### Procedure and center numbers

From 2016 to 2020 a total number of 28,252 LAAC procedures were conducted in Germany. Among them, 28,039 used endocardial occlusion devices and 213 were loop stitch procedures around the LAA base via a pericardial access. While the number of centers performing the endocardial LAA occlusion increased from 311 in 2016 to 384 in 2020, the number of centers performing the epicardial loop stitch procedure around the LAA base was low between 11 and 15 centers (Table 1).

### Patient characteristics

Patient characteristics of all patients from 2016 to 2020 are given in Table 2 and Supplemental Table 2. Patients receiving the endocardial implanted LAA plug were 76.17 ± 8.16 years, 39.06% were female. Cardiovascular and renal comorbidities were common in these patients as well as diabetes and COPD. Patients receiving the epicardial loop stitch around the LAA base were younger (p < 0.001) and less sick according to the Charlson comorbidity index (p = 0.005). According to the CHA_2_DS_2_-VASc Score they had a lower risk of thromboembolic stroke (p = 0.022). They had significantly less of a history of previous myocardial infarctions (7.82% vs. 3.29%, p = 0.014) or previous cardiac surgeries (12.34% vs. 6.57%, p = 0.011). Other comorbidities such as arterial hypertension, heart failure NYHA III or IV, peripheral vascular disease, COPD, pulmonary hypertension, renal disease, diabetes or cancer were similarly distributed in both groups.

### 5-year LAAC procedure number trends

**Fig. 1:**
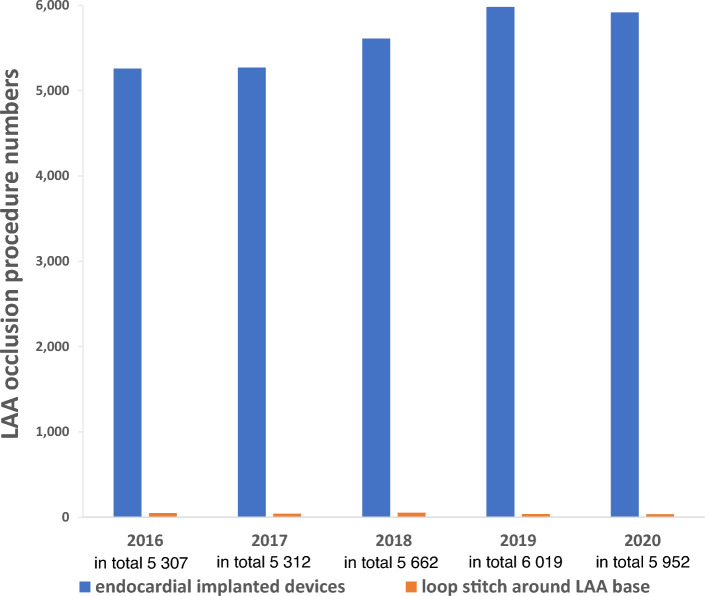
Annual numbers of endocardial implanted LAA occlusion devices and loop stitch procedures around the LAA base in Germany from 2016 to 2020

The 5-year procedure numbers of both the endocardial implanted device and loop stitch procedure increased in total from 5307 in 2016 to 5952 in 2020 (β = 199.7, p = 0.021). The peak was reached in 2019 with 6019 procedures. From 2016 to 2020 annual procedure numbers of the loop stitch around the LAA base did not show a significant trend and remained very rare (lowest procedure number 35 in 2020, highest number 52 in 2018). The annual endocardial implanted LAA plug numbers increased from 5259 in 2016 to 5917 in 2020 (β = 0.203, p = 0.020, Fig. 1) peaking in 2019 with 5981 implantations.

### 5-year patient characteristic trends for endocardial implanted occlusion device

From 2016 to 2020 patient characteristics for the endocardial implanted LAA plugs changed slightly towards older age (β = 0.29, p < 0.001), a higher proportion of female patients (β = 0.46, p = 0.025), more heart failure patients (β = 1.01, p < 0.001) and a higher proportion of patients with renal disease (β = 0.67, p = 0.001) and cancer (β = 0.20, p = 0.001). Patients with a history of COPD (β = − 0.30, p < 0.023) or previous myocardial infarction (β = − 0.23, p = 0.041) became less frequent. Other comorbidities did not show significant trends (Table 3).

**Table 3:** Evolution of baseline characteristics from 2016 to 2020 of patients implanted with an endocardial implanted LAA occlusion device

Patient characteristic endocardial implanted LAAC devices	2016 n = 5259	2017 n = 5271	2018 n = 5610	2019 n = 5982	2020 n = 5917	β per year	p
Age (mean ± SD)	75.53 ± 8.06	75.84 ± 7.95	76.21 ± 8.21	76.52 ± 8.20	76.64 ± 8.28	**0.29**	** < *0.001***
Women	1959 (37.25%)	2093 (39.71%)	2232 (39.78%)	2273 (38.00%)	2394 (40.46%)	**0.46**	***0.025***
Charlson Comorbidity Index	2.22 ± 1.93	2.30 ± 1.96	2.30 ± 1.94	2.31 ± 1.94	2.29 ± 1.90	0.01	ns (0.067)
Arterial hypertension	3145 (59.80%)	3106 (58.93%)	3313 (59.06%)	3518 (58.81%)	3533 (59.71%)	− 0.02	ns
Atrial fibrillation	5152 (97.97%)	5137 (97.46%)	5439 (96.95%)	5785 (96.71%)	5703 (96.38%)	− **0.39**	** < *0.001***
Heart failure	1718 (32.67%)	1830 (34.72%)	1950 (34.94%)	2167 (36.23%)	2189 (37.00%)	**1.01**	** < *0.001***
Heart Failure NYHA III or IV	861 (16.37%)	969 (18.38%)	981 (17.49%)	1121 (18.74%)	1157 (19.55%)	**0.67**	** < *0.001***
Coronary artery disease	2472 (47.01%)	2350 (44.60%)	2564 (45.70%)	2688 (44.93%)	2676 (45.23%)	− 0.32	ns
Previous myocardial infarction	452 (8.59%)	391 (7.42%)	458 (8.16%)	460 (7.69%)	432 (7.30%)	− **0.23**	***0.041***
Previous cardiac surgery	634 (12.06%)	617 (11.71%)	709 (12.64%)	774 (12.94%)	726 (12.27%)	0.16	ns
Peripheral vascular disease	360 (6.85%)	372 (7.06%)	414 (7.38%)	402 (6.72%)	439 (7.42%)	0.08	ns
Carotid disease	87 (1.65%)	85 (1.61%)	101 (1.80%)	110 (1.84%)	98 (1.66%)	0.02	ns
COPD	608 (11.56%)	565 (10.72%)	624 (11.12%)	617 (10.31%)	608 (10.28%)	− **0.30**	***0.023***
Pulmonary hypertension	440 (8.37%)	453 (8.59%)	422 (7.52%)	504 (8.43%)	520 (8.79%)	0.08	ns
Chronic renal disease	1927 (36.64%)	2078 (39.42%)	2165 (38.59%)	2409 (40.27%)	2342 (39.58%)	**0.67**	***0.001***
Chronic renal disease (GFR < 60 ml/min/1.73m^2^)	1680 (31.95%)	1837 (34.87%)	1929 (34.39%)	2163 (36.16%)	2092 (35.36%)	**0.80**	** < *0.001***
Diabetes	1690 (32.14%)	1658 (31.46%)	1711 (30.50%)	1906 (31.86%)	1873 (31.65%)	− 0.04	ns
Cancer	84 (1.60%)	102 (1.94%)	121 (2.16%)	133 (2.22%)	145 (2.45%)	**0.20**	***0.001***

Bold and bold italic highlights significant temporal trends

*LAAC* Left atrial appendage closure, *SD* standard deviation, *NYHA* New York Heart Association, *COPD* Chronic obstructive pulmonary disease

### 5-year safety trends for endocardial implanted LAAC devices

In recent years new endocardial implanted occlusion devices and newer device generations were introduced to clinical practice. Therefore, we analyze the annual safety trends of the LAA plug procedures. Annual numbers of the loop stitch around the LAA base procedure were not sufficient to analyze annual trends.

For endocardial implanted LAAC devices, length of stay, rate of in-hospital mortality, MACCE, pericardial effusion, shock and resuscitation did not change significantly from 2016 to 2020. Only bleeding rates (transfusion > 5 ECs) significantly increased from 1.98% in 2015 to 2.55% in 2020 (β = 0.12, p = 0.050, Table 4). Overall, these data give no sign of in-hospital safety improvements in Germany from 2016 to 2020 for endocardial implanted LAAC device procedures.

**Table 4:** Evolution of safety parameters from 2016 – 2020 of patients implanted with an endocardial implanted LAA occlusion device

Outcome parameters endocardial LAA occlusion device	2016 n = 5259	2017 n = 5271	2018 n = 5610	2019 n = 5982	2020 n = 5917	β per year	p
Length of stay	6.61 ± 7.81	7.10 ± 9.13	7.12 ± 8.81	6.82 ± 8.66	6.99 ± 9.16	0.05	ns
In-hospital mortality	59 (1.12%)	50 (0.95%)	79 (1.41%)	70 (1.17%)	66 (1.12%)	0.02	ns
MACCE	212 (4.03%)	213 (4.04%)	224 (3.99%)	219 (3.66%)	256 (4.33%)	0.02	ns
Myocardial infarction	94 (1.79%)	106 (2.01%)	77 (1.37%)	94 (1.57%)	123 (2.08%)	0.02	ns
Stroke	70 (1.33%)	64 (1.21%)	80 (1.43%)	66 (1.10%)	81 (1.37%)	0.00	ns
Bleeding (transfusion > 5 ECs)	104 (1.98%)	114 (2.16%)	133 (2.37%)	135 (2.26%)	151 (2.55%)	0.12	***0.050***
Intracerebral bleeding	11 (0.19%)	17 (0.32%)	30 (0.53%)	27 (0.45%)	20 (0.34%)	0.04	ns
Pericardial effusion	252 (4.26%)	230 (4.36%)	266 (4.74%)	295 (4.93%)	241 (4.07%)	0.01	ns
Pericardial puncture	93 (1.58%)	114 (2.16%)	109 (1.94%)	131 (2.19%)	107 (1.81%)	0.05	ns
Pericardiotomy	14 (0.23%)	10 (0.19%)	15 (0.27%)	13 (0.22%)	15 (0.25%)	0.01	ns
Deep vein thrombosis	17 (0.29%)	20 (0.38%)	27 (0.48%)	25 (0.42%)	31 (0.52%)	0.05	ns
Tachycardia	176 (2.97%)	128 (2.43%)	157 (2.80%)	189 (3.16%)	143 (2.42%)	− 0.04	ns
Shock	100 (1.69%)	98 (1.86%)	80 (2.48%)	146 (2.44%)	115 (1.94%)	0.10	ns (0.089)
Resuscitation	69 (1.16%)	62 (1.18%)	77 (1.37%)	76 (1.27%)	77 (1.30%)	0.04	ns

Bold italic highlights significant temporal trends

*MACCE* Major adverse cardiac and cerebrovascular events, *EC* erythrocyte concentrate

We conducted further analysis to investigate the association between bleeding and pericardial effusion with death among patients who received an endocardial LAAC device. Patients who experienced major bleeding had a significant higher risk of death compared to those without bleeding (OR 23.34; 95% CI 18.19–29.96%; p < 0.001). Similarly, patients with pericardial effusion also had a significantly higher risk of death (OR 5.93; 95% CI 4.51–7.80%; p < 0.001) (Supplemental Tables 3, 4).

### Center volume dependent outcomes of endocardial implanted LAAC devices

We then determined whether the number of endocardial LAAC procedures performed by a single center was associated with in-hospital MACCE and pericardial puncture. Our analysis revealed that neither MACCE (p = 0.083) or pericardial puncture (p = 0.080) showed any dependency on center volumes.

### In-hospital safety comparison

Total numbers and relative numbers of in-hospital safety parameters regarding in-hospital mortality, MACCE, bleeding, pericardial effusion, pericardial puncture, shock and resuscitation of patients receiving the endocardial implanted LAAC device and loop stitch around the LAA base from 2016 to 2020 are given in Table 5. To compare both types of LAAC procedures we used a propensity score approach. Length of hospital stay was similar in both groups. In-hospital mortality and in-hospital MACCE showed no significant difference between the endocardial LAA plug procedure and the epicardial loop stitch procedure.

**Table 5:** Safety parameters while hospitalization for endocardial implanted LAAC procedure and epicardial loop stitch around the LAA base procedure from 2016 – 2020

Safety parameters while hospitalization	Total procedures n = 28,252	endocardial implanted LAA occlusion device n = 28,039	loop stitch around the LAA base n = 213
Length of stay (days, mean ± SD)	6.93 ± 8.72	6.93 ± 8.74	7.33 ± 6.58
In-hospital mortality	326 (1.15%)	325 (1.16%)	1–3 (≤ 1.41%)
MACCE	1130 (4.00%)	1124 (4.01%)	6 (2.82%)
Myocardial infarction	496 (1.76%)	493 (1.76%)	3 (1.41%)
Stroke	365 (1.29%)	362 (1.29%)	3 (1.41%)
Bleeding (transfusion > 5 ECs)	639 (2.26%)	637 (2.27%)	1–3 (≤ 1.41%)
Intracerebral bleeding	104 (3.68%)	104 (0.37%)	0 (0.00%)
Pericardial effusion	1289 (4.56%)	1256 (4.48%)	33 (15.49%)
Pericardial puncture	572 (2.02%)	544 (1.94%)	28 (13.15%)
Pericardiotomy	67 (0.24%)	64 (0.23%)	3 (1.41%)
Deep vein thrombosis	120 (0.42%)	118 (0.42%)	1–3 (≤ 1.41%)
Tachycardia	795 (2.81%)	774 (2.76%)	21 (9.86%)
Shock	589 (2.08%)	586 (2.09%)	1–3 (≤ 1.41%)
Resuscitation	355 (1.26%)	353 (1.26%)	1–3 (≤ 1.41%)

*LAA* Left atrial appendage, *SD* standard deviation, *MACCE* Major adverse cardiac and cerebrovascular events (in-hospital mortality/myocardial infarction/stroke), *EC* erythrocyte concentrate

**Fig. 2:**
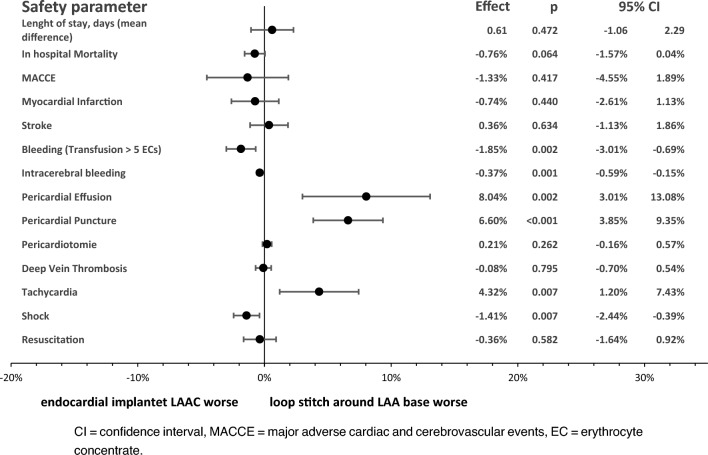
Forest plot of propensity score approach to compare adjusted risks of in-hospital safety parameters. *CI* confidence interval, *MACCE* major adverse cardiac and cerebrovascular events, *EC* erythrocyte concentrate

Risk of transfusion with more than 5 red blood cell concentrates (− 1.85%; 95% CI − 3.01 to − 0.69%; p = 0.002), intracerebral bleeding (− 0.37%; 95% CI − 0.59 to − 0.15%; p = 0.001) and shock (− 1.41%; 95% CI − 2.44 to − 0.39%; p = 0.007) was significantly lower for the loop stitch procedure, while risk of pericardial effusion (− 8.04%; 95% CI 3.01 to 13.08%; p = 0.002), pericardial puncture (6.60%; 95% CI 3.85 to 9.35%; p < 0.001) and tachycardia (− 4.32%; 95% CI 1.20 to 7.43%; p = 0.007) was significantly lower for the endocardial implanted occlusion device procedure. In-hospital risk of myocardial infarction, stroke, deep vein thrombosis and resuscitation did not show significant differences between both LAA procedure types (Fig. 2).

## Discussion

In this real-world analysis of 28,252 patients included we present a nation-wide dataset of procedure numbers and safety trends of catheter based LAA closure procedures for all patients treated in Germany from 2016 to 2020. An endocardial implanted LAA occlusion device was the most frequently used LAAC procedure in this period in Germany while a loop stitch around the LAA base was done rarely. Safety parameters did not show significant trends for the endocardial implanted occlusion device from 2016 to 2020 except of an increase in bleeding rates.

In-hospital mortality and in-hospital MACCE did not show significant differences between both procedures while the loop stitch around the LAA procedure was associated with more tachycardia, pericardial effusion and pericardial punctures and the endocardial implanted LAA occlusion device procedure with more bleeding, intracerebral bleeding and shock.

The lack of any signal for an improvement in procedural safety is somewhat surprising. Newer generations of endocardial LAA occlusion devices were introduced in the clinical routine from 2013. The second generation Watchman FLX received a first CE mark in 2015 but was retracted from the market and has got a new CE mark since 2019. The second generation Watchman FLX (Boston Scientific) finally received the CE mark in 2019. Amulet (Abbott), the second generation Amplatzer occluder, has got a CE mark since 2013. Other newer occluders like WaveCrest (Biosense) or Occlutech (Occlutech) received the CE mark in 2013 and 2016, respectively [20].

The second generation Watchman device had low procedural complication rates in a multicenter registry with 165 patients [24] similar to a randomized multicenter industry sponsored trial comparing the first and second Watchman generations which found a very low incidence of adverse events for both generations [25]. Clinical outcomes at 45 days did not differ between Amulet and Watchman in the randomized multicenter industry supported SWISS APERO trial with 221 patients. This study did also not find clear evidence that the newer Watchman FLX provides superior LAA sealing compared with the earlier-generation [26]. The data of the here presented study show no signal of in-hospital safety improvements in a 5-year period after the introduction of new LAA occluder generations. In fact, in-hospital safety remained on a similar level as reported in an earlier real-world study by Hobohm et al. [12], who investigated safety trends for LAA occlusion in Germany from 2011 to 2015. Total procedure numbers increased from 1347 in 2011 to 4932 in 2015 to 5917 in 2020. Most patient characteristics were similar in the 2016 to 2020 and 2011 to 2015 cohort, with the exception of chronic renal disease, which was evident more frequently in the 2016 to 2020 cohort. Safety trends for the endocardial implanted device from 2016 to 2020 remained similar compared with the cohort from 2011 to 2015: In-hospital mortality remained in a range from 0.9 to 1.4% from 2013 to 2020. MACCE was similar in both studies. Also, pericardial effusion and pericardial puncture rates in our endocardial implanted occlusion device group were similar to the 2011 to 2015 data. Similar invasive procedures such as atrial fibrillation ablation are associated with comparable pericardial effusion rates of 1 to 6% [12, 27, 28].

The overall rate of transfusion > 5 ECs after LAAC in Germany was 2.26% from 2016 to 2020. Newer short term analyses of bleeding rates after Watchman FLX implantation report approximately 3% [24, 25]. The SWISS APERO trial reported major bleeding of 7.2% for the Amulet device and 1.8% for the Watchman occluder at 45 days after implantation [26]. A Canadian registry reported 4.7% major bleeding implantation within 7 days after Watchman implantation [8], a European registry reported 0.9% major bleeding within 7 days after Watchman implantation [11]. Another real world analysis of LACC in Germany from 2011 to 2015 reported transfusion rates of 10% [12]. Reasons for different bleeding rates might be heterogenous patient populations, different definitions of (major) bleeding and reporting time frames.

The endocardial implanted LAAC procedure was preferred in Germany from 2016 to 2020. One reason might be a longer history of experience and training with this type of occluder. Another possible reason could be the higher complexity of the invasive loop stitch technique which requires a combination of transseptal puncture and pericardial access.

ESC guidelines state that for patients who do not tolerate any antiplatelet therapy, an epicardial catheter approach (e.g. Lariat) may be an option [1, 29, 30]. Our data show, that the loop stitch procedure was associated with a significantly lower risk of bleeding. In contrast to the endocardial implanted LAA occlusion devices, which are usually treated with single or dual antiplatelet therapy for a period of several months [6, 24], the epicardial catheter approach usually does not need antiplatelet therapy. Our analysis supports the recommendation that patients with high bleeding risk from antiplatelet therapy can be considered for the epicardial loop stitch procedure.

To our knowledge, this is the first study comparing in-hospital safety of the widely used endocardial implanted plug occlusion devices and a loop stitch procedure around the LAA base in a head-to-head fashion. This real world data can be regarded as free of study selection bias, as all patients reimbursed in Germany were analyzed. More pericardial effusion and necessity of pericardial puncture for the LAA loop stitch procedure is likely caused by pericardial puncture during the procedure. Conversely, higher rates of bleeding and shock were present in patients implanted with an endocardial LAAC device.

The Lariat device is the only currently available epicardial LAAC procedure. Few reports about Lariat safety exist but none of them compares the Lariat device with other procedure types [31]. These studies report heterogenous results. In a multicenter study by Parik et al. with 108 patients implanted with Lariat no procedure related mortality was evident but 1.09% of the patients needed open heart surgery, 2.8% had cardiac perforation without the need of surgery and 1.9% needed transfusion [32]. Another multicenter trial by Tilz et al. with 141 patients reported a serious adverse event rate of 2.8% [33]. A study by Fink et al. with 44 patients undergoing successful LAA ligation with Lariat found an incidence of major periprocedural complications of 6%, and a significant rate of incomplete LAA ligation during follow-up. They suggest that epicardial LAA ligation should only be performed in selected patients and in centers with experience in dry epicardial puncture and on-site cardiac surgery [34, 35]. Overall, due to the technically challenging procedure and complications it was believed to be unlikely that Lariat would capture market share compared with endocardial occlusion systems. However, refining the technique and new benefits of LAA ligation made the community reconsider this [18]. In hypertensive AF patients, epicardial LAA occlusion significantly decreased systolic blood pressure compared with endocardial LAAC [36]. A prospective, multicenter, randomized controlled trial was initiated to evaluate the safety and effectiveness of the Lariat system to percutaneously isolate and ligate the left atrial appendage from the left atrium as an adjunct to pulmonary vein isolation in the treatment of subjects with symptomatic persistent or longstanding persistent atrial fibrillation (aMAZE, ClinicalTrials.gov Identifier: NCT02513797). This trial showed that LAA ligation with Lariat plus pulmonary vein isolation was not superior at preventing recurrent atrial fibrillation compared with pulmonary vein isolation alone. Exploratory analysis of this study suggested that possible benefits among patients with early persistent atrial fibrillation and large left atrial volumes, which both needs to be further investigated.

We did not particularly investigate the influence of the COVID-19 pandemic [37–39] starting in January 2020 on the LAA occlusion numbers and patient selection in Germany [40]. In 2020 the total procedure numbers slightly decreased to 5917 from a high of 6019 procedures in 2019. Further analysis of the period from 2020 to 2022 is warranted to clarify COVID-19 impact. Data on this will be expected in 2024.

### Study limitations

Our study has several limitations. First, the ICD discharge code based data collection used for our analysis is potentially biased by under- or overreporting. It is very unlikely that the here used end-points like in-hospital MACCE were under-reported, as an increase in resource-utilization often triggers an additional reimbursement. It is also not reported at what time of the hospital stay an outcome event occurred or if it’s associated with a procedure other than LAAC of the same hospital stay. In the German reimbursement system it is unlikely that a second major procedure (e.g. coronary angiography) is carried out during the same hospitalization as LAAC. Second, we cannot address long-term outcomes with ICD discharge codes. Third, we cannot distinguish between different endocardial implanted occlusion devices and indications that all use the same OPS-code. Fourth, the HAS BLED score cannot be calculated with ICD codes [41, 42]. Fifth, the entire endpoint set from the Munich consensus document for LAAC cannot be covered with ICD and OPS codes and had therefore be simplified [43]. Last, a selection bias for the loop stitch procedure patients is likely and limit the comparison to the large cohort treated with the endocardial devices.

## Conclusions

In Germany, numbers of catheter based LAAC increased from 5307 in 2016 to 5952 in 2020. In the majority of cases the catheter based LAAC was performed with an endocardial implanted LAA occlusion device. An epicardial loop stitch procedure around the base of the LAA was carried out rarely. The LAA loop stitch procedure was associated with higher risk of pericardial effusion and pericardial puncture while the plug procedure was associated with a higher risk of bleeding, intracerebral bleeding and shock. Our analysis supports the recommendation that patients with a high bleeding risk from antiplatelet therapy can be considered for the epicardial loop stitch procedure, but that this should be reserved for these very selected cases. Furthermore, these data show no sign of in-hospital safety improvements for endocardial implanted LAAC devices from 2016 to 2020.

## Supplementary Information

Below is the link to the electronic supplementary material.

Supplementary file1 (DOCX 350 KB)

## Abbreviations

LAA: Left atrial appendage
LAAC: Left atrial appendage closure
OAC: Oral anticoagulation
CE: Conformité Européenne
MACCE: Major adverse cardiac and cerebrovascular events
COPD: Chronic obstructive pulmonary disease
EC: Erythrocyte concentrate
CI: Confidence interval
NYHA: New York Heart Association
