# Supplementary material for: Catheter based left atrial appendage closure in-hospital outcomes in Germany from 2016 to 2020
Source: Clin Res Cardiol. 2023 Sep 12;113(10):1419–29. doi: 10.1007/s00392-023-02299-w (PMC11420385; doi:10.1007/s00392-023-02299-w)
Supplement: Supplementary file 1 — Supplementary file1 (DOCX 350 KB) [file 392_2023_2299_MOESM1_ESM.docx]

**SUPPLEMENTAL**

**Supplemental Table 1**

| **Characteristic** | **Total procedures**  **n = 28 252** | **endocardial implanted LAA occlusion device**  **n = 28 039** | **loop stitch around the LAA base**  **n= 213** | **p** |
| --- | --- | --- | --- | --- |
| CHA_2_DS_2_-VASc 0 | 198 (0.70 %) | 196 (0.70 %) | 2 (0.94 %) |  |
| CHA_2_DS_2_-VASc 1 | 924 (3.27 %) | 914 (3.26 %) | 10 (4.96 %) |  |
| CHA_2_DS_2_-VASc 2 | 2 931 (10.37 %) | 2 904 (10.36 %) | 27 (12.68 %) |  |
| CHA_2_DS_2_-VASc 3 | 5 803 (20.54 %) | 5 748 (20.50 %) | 55 (25.82 %) |  |
| CHA_2_DS_2_-VASc 4 | 7 803 (27.62 %) | 7 755 (27.66 %) | 48 (22.54 %) |  |
| CHA_2_DS_2_-VASc 5 | 6 606 (23.38 %) | 6 561 (23.40 %) | 45 (21.13 %) |  |
| CHA_2_DS_2_-VASc 6 | 3 182 (11.26 %) | 3 160 (11.27 %) | 22 (10.33 %) |  |
| CHA_2_DS_2_-VASc 7 | 698 (2.47 %) | 695 (2.48 %) | 3 (1.41 %) |  |
| CHA_2_DS_2_-VASc 8 | 96 (0.34 %) | 95 (0.34 %) | 1 (0.47 %) |  |
| CHA_2_DS_2_-VASc 9 | 11 (0.04 %) | 11 (0.04 %) | 0 (0 %) |  |
| Any surgery while hospitalization | 3 202 (11. 33 %) | 3 169 (11.30 %) | 33 (15.49 %) | ns (0.05459) |

**Supplemental Table 2**

| **Patient characteristic endocardial implanted LAAC devices** | **2016**  **n = 5 259** | **2017**  **n = 5 271** | **2018**  **n = 5 610** | **2019**  **n = 5 982** | **2020**  **n = 5 917** | **ß per year** | **p** |
| --- | --- | --- | --- | --- | --- | --- | --- |
| Any Surgery while hospitalization | 571  (10.86 %) | 572  (10.85 %) | 634  (11.35 %) | 652  (10,90 %) | 737  (12.46 %) | **0.33** | ***0.014*** |

**Supplemental Table 3**

|  |  | **Bleeding** | |  |  |
| --- | --- | --- | --- | --- | --- |
|  |  | 0 | 1 |  | Total |
| **Death** | 0 | 27,180 | 535 |  | 27,715 |
|  | 1 | 222 | 102 |  | 324 |
|  | Total | 27,402 | 637 |  | 28,039 |
|  |  |  |  |  |  |
|  | **OR** |  | **p** | **95 % CI** | |
|  | 23.34 |  | 0.000 | 18.19 | 29.96 |

**Supplemental Table 4**

|  |  | **Pericardial effusion** | |  |  |
| --- | --- | --- | --- | --- | --- |
|  |  | 0 | 1 |  | Total |
| **Death** | 0 | 26 527 | 1 188 |  | 27 715 |
|  | 1 | 256 | 68 |  | 324 |
|  | Total | 26 783 | 1 256 |  | 28 039 |
|  |  |  |  |  |  |
|  | **OR** |  | **p** | **95 % CI** | |
|  | 5.93 |  | 0.000 | 4.51 | 7.80 |
